# Biomarkers for Endometrial Receptivity: Implications for Infertility, Implantation Failure, and Advances in Diagnosis and Treatment

**DOI:** 10.3390/ijms27104209

**Published:** 2026-05-09

**Authors:** Vidhu Dhawan, Nazia Sunny, Filomena Mottola, Ilaria Palmieri, Lorenzo Ibello, Rima Dada, Jogen C. Kalita, Israel Maldonado Rosas, Shubhadeep Roychoudhury

**Affiliations:** 1 Department of Anatomy, All India Institute of Medical Sciences (AIIMS), New Delhi 110029, India; 2 Department of Life Science and Bioinformatics, Assam University, Silchar 788011, India; 3 Department of Environmental, Biological and Pharmaceutical Sciences and Technologies, University of Campania Luigi Vanvitelli, 81100 Caserta, Italy; 4 Department of Zoology, Gauhati University, Guwahati 781014, India; 5 Citmer Reproductive Medicine, Mexico City 11520, Mexico

**Keywords:** reproductive health, endometriosis, cytokines, implantation window (WOI), pregnancy loss, embryo implantation, implantation biomarkers

## Abstract

Female infertility affects millions of women worldwide and is frequently caused by ovulatory disorders and uterine pathologies. Among these, endometrial abnormalities play a central role, especially in cases of embryo implantation failure during assisted reproductive techniques (ARTs). Endometrial receptivity, the period during which the endometrium prepares to receive and support embryo implantation, is a complex process regulated by essential morphological, molecular, and immunological changes necessary for a successful pregnancy. This review examines the mechanisms that govern endometrial receptivity and investigates the dysfunctions that may compromise this phase. In particular, it focuses on key biomarkers of receptivity, such as estrogen receptor-alpha (ERα), integrins, and uterine immune cells, which are crucial for endometrial preparation. It provides an in-depth understanding of female reproductive physiology and the main indicators to optimize embryo transfer at the most opportune time during the menstrual cycle within medically assisted reproductive techniques. Furthermore, it explores innovative approaches, such as immunomodulation and mesenchymal stem cell therapy, which are opening new diagnostic and therapeutic horizons, offering hope to many couples facing reproductive challenges.

## 1. Introduction

Reproduction is of prime importance in the advancement of human society. Recent data shows a significant 40% decrease in the average number of children per woman over the past four decades, indicating notable shifts in demographic patterns [1]. Globally, 8–12% of couples face infertility issues, with malfunctions in the endometrium being a predominant factor [2]. Between 1990 and 2017, the annual increase in female infertility prevalence reached 0.37%, surpassing the 0.291% rise observed in men [3]. Recurrent implantation failure (RIF) is among the prime causes of female infertility. The European Society of Human Reproduction and Embryology (ESHRE) Working Group on RIF recommends considering RIF as a secondary phenomenon of assisted reproductive technology (ART), observed only in in vitro fertilization (IVF) patients. To clarify the definition, they suggest adopting the following description: “RIF refers to the situation where the transfer of viable embryos has failed to result in a positive pregnancy test often enough in a specific patient to justify further investigations and/or interventions”. The probability of successful implantation after ART depends on various factors, including age, hormones, uterine status, embryo quality, genetic disorders, and the quality of the laboratory. Although there is no perfect model, existing data, such as those from the European IVF Monitoring Program, can be used to estimate the likelihood of success [4].

Given the various causes of RIF, there is a growing focus on preserving the cyclic repair of the endometrium [5].

The human endometrium, a highly adaptable lining of the uterus, undergoes a cyclical process throughout the menstrual cycle, involving shedding, repair, and regeneration, which repeats monthly during a woman’s reproductive years. After childbirth, the endometrium fully restores itself without scarring, similar to fetal wounds and certain adult mucosal tissues. It regrows to its original thickness, crucial for embryo implantation and placental establishment in subsequent cycles. A key phase in this process is the implantation window or window of implantation (WOI), during which the endometrium becomes optimally receptive for embryo implantation, which is pertinent for successful pregnancy [6].

Endometrial receptivity, a crucial factor in the process of successful embryo implantation, unfolds through intricate molecular and cellular mechanisms. The clinical implications of understanding endometrial receptivity are profound, especially in infertility, recurrent pregnancy loss (RPL), RIF, and conditions such as endometriosis. Traditionally, endometrial histology serves as the primary measure of receptivity, but its limitations, especially in women with infertility, necessitate more nuanced approaches. Integrin testing has emerged as a valuable tool, revealing that the absence of the beta 3 (β3) integrin subunit is associated with receptivity defects, notably in cases of infertility and RPL. Moreover, aberrant β3 integrin expression has been observed in women with endometriosis, underscoring its clinical relevance [7]. Treatments such as salpingectomy or aromatase inhibitor therapy have shown promise in restoring both fertility and integrin expression levels. Notably, the association between β3 integrin expression and downregulation of ERα further underscores the significance of understanding endometrial receptivity in managing conditions like luteal phase defect and endometriosis [8].

This analysis carefully examines these mechanisms, delving into a thorough exploration of the intricate landscape that characterizes the receptivity of the endometrium during implantation [9]. The landscape of reproductive health broadens further as the impact of inflammation and injury on the endometrium is scrutinized. The scrutiny extends to the roles of cytokines, uterine natural killer (uNK) cells, tumour necrosis factor (TNF), and other factors in endometrial inflammation. Additionally, the practice of endometrial scratching is used in ARTs. Its potential impact on implantation success is highlighted [6]. In the transition to therapeutic approaches, this data-driven study examines emerging strategies designed to address endometrial issues and enhance reproductive outcomes. At the forefront is immunomodulation, a focal point in the discourse, as the potential to amplify endometrial receptivity and alleviate inflammation-related infertility is explored. Further into the frontier, recent advancements in mesenchymal stem cell therapy for endometrial regeneration are explored, envisioning a future direction in reproductive medicine [10].

The discussion extends beyond the laboratory setting to encompass the clinical realm, exploring the implications of impaired endometrial receptivity. Conditions such as infertility, pregnancy loss, endometriosis, and polycystic ovary syndrome (PCOS) are addressed. With a focused lens on disruptions in endometrial receptivity within the context of endometriosis and PCOS, the association with RIF and pregnancy loss is underscored [11].

Within this framework, endometrial biomarkers have emerged as key tools for assessing the receptive state of the endometrium and for identifying the optimal WOI. Advances in molecular and transcriptomic profiling, including targeted gene expression analyses, have provided new insights into the complex regulatory mechanisms underlying endometrial receptivity and have led to the development of commercially available diagnostic approaches [6,10].

Despite extensive research and numerous published reviews, the clinical translation of these biomarkers remains limited. To date, no single biomarker has demonstrated sufficient accuracy and reproducibility to be routinely adopted in clinical practice. Even recent genomic and transcriptomic approaches are supported by heterogeneous and often inconclusive evidence. Major limitations include the lack of methodological standardization, population heterogeneity, limited reproducibility across studies, and the absence of large-scale prospective validation [12]. These issues are particularly relevant for commercially available tests aimed at defining the WOI, which are increasingly used in ART despite insufficient evidence to support their routine application [12].

In light of these challenges, this review aims to provide a critical appraisal of currently available endometrial receptivity biomarkers, focusing on the translational gap between biological discovery and clinical implementation. Specifically, it evaluates the strength of biological and clinical data supporting each biomarker, distinguishes established markers from those still at an exploratory stage, and discusses future strategies to facilitate their integration into clinical practice [13].

## 2. Implications for Infertility and Implantation

### 2.1. Endometrial Receptivity

Endometrial receptivity is a crucial aspect of female reproductive physiology, characterized by the endometrium’s ability to support embryo implantation. The endometrium is a fundamental layer of the female reproductive system, as it provides an ideal environment for the development of the zygote. This tissue forms the inner lining of the uterus and, in addition to its crucial role during pregnancy, the endometrium undergoes cyclical renewal during the menstrual cycle, underscoring its centrality to fertility and reproductive health. This environment is regulated through interactions with the ovaries, which control the production of ovarian follicles and estrogen levels. These processes guide the cyclical repair and regeneration of the endometrium, ensuring its receptivity and allowing successful implantation [14].

Cyclic endometrial repair and regeneration are closely linked to the female reproductive system, where the endometrium plays a central role. This categorization divides cyclical endometrial repair and regeneration into three scenarios: regular menstrual cycle repair, deficient repair, and excessive repair [15]. As is well known, in women of reproductive age, the growth of ovarian follicles leads to ovulation and the formation of the corpus luteum, which cyclically secretes estrogen and progesterone. If the embryo fails to implant, hormone levels drop, leading to a reduction in blood flow to the endometrium. This triggers ischemia, tissue damage, and menstrual bleeding. Remarkably, the endometrium undergoes repair during menstruation without forming scars, thanks to rapid re-epithelialization, a process supported by inflammation and the activation of immune cells [6,16].

The menstrual phase and the subsequent repair process of the endometrium are governed by a series of cellular and molecular events involved in tissue removal and the preparation of the endometrial microenvironment for potential implantation. Given that inflammation and extracellular matrix remodeling are key elements in regulating endometrial receptivity, the proteins involved in these processes could provide valuable information about the phase of the cycle the endometrium is in and its suitability to accommodate the embryo, making them potential indicators of receptivity.

Since 1989, research has proposed the presence of stem cells in the basal layer of the endometrium, crucial for cyclic endometrial regeneration. Recent studies have identified various stem-like cells in the endometrium, including epithelial progenitors and endometrial mesenchymal stem cells (eMSCs), capable of self-renewal and differentiation in vitro. Notably, eMSCs, particularly when carried on scaffolds, have shown potential to accelerate endometrial repair processes. Earlier investigations in the 1980s highlighted estrogen’s role in stimulating basal glandular cells, promoting their proliferation and migration into the endometrial cavity. Furthermore, estrogen has been linked to vascular regeneration [15]. However, the intricate molecular mechanisms governing both endometrial regeneration and repair remain areas of ongoing research, necessitating further exploration to fully understand their complexities. During the proliferative phase, estrogen stimulates the growth and fortification of the endometrium, while in the secretory phase, progesterone and estrogen promote the structural and functional changes necessary for embryo adhesion. Stromal cells transform into pre-decidual cells to support implantation. The complexity of embryo implantation involves intricate interactions between the embryo and the maternal endometrium over approximately 4–6 days during the mid-luteal phase [17]. It is a crucial step in establishing a successful pregnancy, where a fertilized embryo attaches and embeds within the endometrium. This attachment usually occurs during the blastocyst stage, around 5–7 days after fertilization. Trophoblast cells of the blastocyst adhere to and embed into the endometrial tissue, initiating placenta formation and supporting early pregnancy [18]. This phase corresponds to the mid-secretory phase from Day 19 to Day 23 of the menstrual cycle, known as the WOI [19]. This interaction involves the apposition, adhesion, and invasion of the blastocyst into the endometrium. The embryo is resistant to successful implantation and establishment of pregnancy during any other phase of the menstrual cycle [20]. Dysfunctions in these processes, such as poor repair or defective transformation into decidua, can compromise receptivity, leading to implantation difficulties and infertility.

Optimal receptivity of the endometrium is vital for successful implantation and subsequent pregnancy establishment, posing challenges in ARTs such as IVF. Despite advancements, factors such as embryo quality and endometrial receptivity continue to influence implantation outcomes [20]. During WOI, crucial events that unfold include changes in the endometrium to facilitate embryo implantation, molecular and cellular alterations to promote embryo attachment, and synchronization between embryo development and the receptive endometrium. Successful synchronization and implantation signify the establishment of pregnancy. The identification of the precise time and period of endometrial receptivity is thus the cornerstone of the successful outcome of any assisted reproductive cycle [21].

Certain inflammatory or anatomical conditions can alter or narrow this window, leading to challenges in normal implantation, infertility, or pregnancy loss. Immune cells, including dendritic cells, macrophages, neutrophils, T cells, B cells, and natural killer (NK) cells, play a critical role in regulating endometrial receptivity. Among these, uNK cells are particularly significant. They are the predominant lymphocytes found in the decidua during implantation and the early stages of pregnancy, where they contribute to the successful embryo implantation process [22].

#### Factors Influencing Endometrial Receptivity

**Figure 1:**
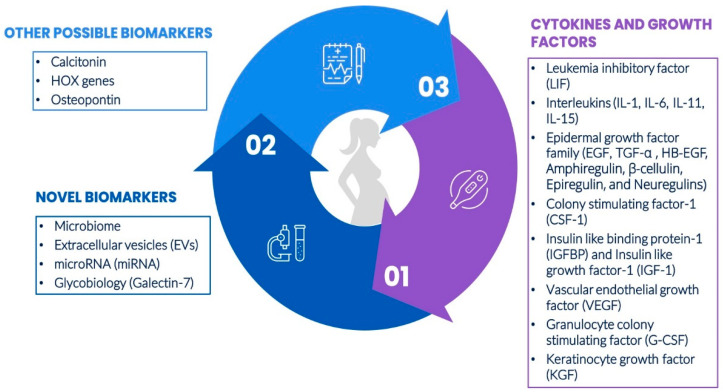
A comprehensive outline of various endometrial biomarkers of infertility.

Endometrial receptivity is a complex process influenced by multiple biological and pathological factors. Among the main factors that can compromise this receptivity are endometrial inflammation, endometriosis, and PCOS. These conditions, by creating an inflammatory uterine environment, can interfere with the WOI, reducing the chances of a healthy pregnancy. These factors, interacting with endometrial biomarkers and immune mechanisms, play a crucial role in the success of fertility treatments and in achieving pregnancy (Figure 1).

Endometrial inflammation, characterized by infection or inflammation of the endometrium, can manifest in acute or chronic forms. Typically, devoid of microorganisms, the endometrium may become susceptible to bacterial migration from the cervix and vagina, leading to inflammation and infection. Postpartum endometritis, often linked with preterm pre-labour rupture of membranes (PPROM), is more prevalent in cesarean sections and commonly involves various bacterial strains. Chronic endometritis, frequently asymptomatic, is often identified during assessments for secondary amenorrhea and infertility [23]. On the other hand, endometrial injury involves intentionally damaging the endometrium to enhance reproductive outcomes. The primary method, endometrial scratching, typically employs a pipelle to obtain a sample, triggering a healing response aimed at improving endometrial receptivity and boosting the likelihood of successful implantation and pregnancy [13].

Histopathologically, acute endometritis is characterized by microabscesses within the endometrium and the presence of neutrophils in the surface epithelium and glandular lumens. Clinically, it manifests symptoms including fever, pelvic pain, heightened vaginal discharge with abnormal odour, consistency and colour, abdominal discomfort and bloating, irregular vaginal bleeding, altered bowel movements, and overall fatigue [23].

Acute infections may arise from both aerobic and anaerobic bacteria. Following a cesarean section, endometritis commonly results from infections caused by Streptococcus pyogenes and Staphylococcus aureus. Chlamydia-induced endometritis typically presents later, about 7 days after delivery. Examples of aerobic bacteria include Group A Streptococcus, Group B Streptococcus, Staphylococcus, and *Escherichia coli*. Examples of anaerobic bacteria include Peptostreptococcus, Peptococcus, and Bacteroides [24].

Chronic endometritis, or CE, is a condition characterized by prolonged and mild inflammation of the endometrial lining, often accompanied by plasma cell infiltration into the stromal area. Its prevalence is underestimated due to diagnostic challenges, with rates varying widely from 0.2% to 46% depending on patient characteristics and the diagnostic methods used. Research suggests that 45% of infertile patients, particularly those with RIF, may have CE. Histopathological features include oedema in the superficial endometrial lining, increased stromal cell density, asynchronous maturation between stroma and epithelium, and infiltration of plasma cells, specifically endometrial stromal plasma cells (ESPCs). The presence of numerous ESPCs is considered the most sensitive and specific indicator for diagnosing and defining CE [23].

CE patients present symptoms like pelvic discomfort, spotting, and abnormal vaginal discharge (leucorrhoea). Patients may also report lighter than usual menstrual flow (hypomenorrhoea), absence of periods (secondary amenorrhoea), and difficulty conceiving (infertility) [23]. Common microbes associated with endometritis include *E. coli*, Streptococcus, Enterococcus, Staphylococcus, *Mycoplasma* spp., Ureaplasma urealyticum, Gardnerella vaginalis, Proteus, Klebsiella pneumoniae, Pseudomonas aeruginosa, Corynebacterium, yeasts such as Saccharomyces, *Candida* spp., and Mycobacterium tuberculosis. It is noteworthy that while acute endometritis is typically linked with pathogens like Chlamydia trachomatis and Neisseria gonorrhoeae, these are rarely found in CE cases, indicating that the microbial profiles of acute and chronic endometritis are distinct [23].

CE is a condition that can compromise endometrial receptivity in the context of endometriosis. As a chronic, immune-mediated inflammatory disease, it may be associated with an abnormal uterine microenvironment. In this environment, microbial inflammation occurs in the uterine cavity, with the presence of lymphocyte subpopulations that cause altered secretion of paracrine factors. This may reduce the endometrium’s ability to support embryo implantation, thereby compromising endometrial receptivity [25].

Endometriosis and adenomyosis both involve the presence of endometrial tissue outside the uterus, but they affect fertility and pregnancy differently. These conditions often coexist with other reproductive disorders, like fibroids, complicating their impact on fertility outcomes. Endometriosis, characterized by ectopic endometrial tissue that causes inflammation and pain, is estrogen-dependent and a chronic condition. It can contribute to infertility through various mechanisms, though no single cause is definitive. In severe cases, women may have trouble conceiving, RPL, or both, despite initially being able to conceive. Around 40% of women with infertility are affected by endometriosis, and it is associated with poorer ART outcomes, including reduced egg yield, lower embryo implantation rates, and decreased pregnancy success.

Endometriosis can also cause ovarian damage and follicular loss, as well as abnormalities in the endometrium, which may affect embryo implantation. Additionally, toxic pelvic factors can negatively impact oocyte quality, increasing the risk of aneuploidy, especially in natural conception cycles. Inflammation in the eutopic endometrium can impair pre-decidual transformation and placental development, contributing to early pregnancy losses and RPL. Studies have shown that endometriosis disrupts implantation timing, further raising the risk of miscarriage and RPL due to altered progesterone-driven changes in the endometrium.

Acknowledged increasingly as a societal concern, infertility often ties back to conditions like PCOS, known for its profound impact. PCOS arises from intricate immune-metabolic pathways, ultimately leading to reproductive challenges. Within PCOS, infertility primarily arises from issues with ovulation and implantation, spurred by mild inflammation affecting ovarian and endometrial tissues. This inflammation, fueled by immune and metabolic imbalances, is intertwined with systemic responses and disturbances, such as insulin resistance, hypoadrenalism, progesterone insufficiency, and oxidative stress. These factors not only contribute to various health ailments like cardiovascular diseases, cancer, and autoimmunity but also exert significant repercussions on fertility [26].

Women with PCOS show an alteration in hormone levels, with an elevated production of luteinizing hormone (LH) and a reduction in follicle-stimulating hormone (FSH), which hinders the ovulation process. This imbalance between LH and FSH prevents the proper maturation of follicles. In addition, it is common for women with PCOS to have higher levels of anti-Mullerian hormone (AMH), produced by immature follicles. AMH levels reflect the blockage of follicular development and are closely linked to the severity of PCOS symptoms. This hormonal dysfunction leads to the blockage of follicular maturation, the absence of ovulation, and irregular or completely absent menstrual cycles. Unlike PCOS, women with endometriosis have a reduced ovarian reserve associated with lower AMH levels than healthy women, with an inverse relationship between AMH levels and disease severity. In women with endometriosis, granulosa cells in the antral follicles have a low concentration of LH receptors throughout the menstrual cycle. This results in longer menstrual cycles, characterized by more prolonged follicular phases and a delay in the rise in LH levels [27].

### 2.2. Endometrial Receptivity Biomarkers

Achieving successful implantation in assisted reproduction in women is a significant challenge, with success rates typically ranging from 30–40% [28]. This success hinges on effective communication between the embryo and the receptive endometrium, with numerous molecular factors being expressed during the receptive phase. These factors, including growth factors, cytokines, calcitonin, homoeobox (HOX) genes, and cell adhesion molecules, are crucial for implantation. Cytokines, functioning as chemical messengers, can indicate uterine receptivity. Understanding these biomarkers and their role in determining the WOI is crucial for diagnozing and treating infertility more effectively in IVF couples, potentially leading to improved outcomes in terms of successful implantation and subsequent pregnancy [29]. An outline of the endometrial biomarkers of female infertility is provided in Figure 1.

During the brief period of WOI, uterine receptivity is at its peak for blastocyst implantation. Furthermore, WOI is marked by changes in the morphology of the endometrial epithelium, including the emergence of pinopods [30]. Pinopods are transient protrusions of endometrial epithelial cells that appear during the WOI and are considered a morphological indicator of endometrial receptivity. These structures, morphologically similar to domes, appear to facilitate the initial interaction between the endometrium and the blastocyst, contributing to its adhesion and retention [31].

These are structures dependent on progesterone, appearing as cellular protrusions on the endometrial surface around Days 20–21 of the menstrual cycle [32]. Detection of their expression via scanning electron microscopy (SEM) in sequential endometrial biopsies can serve as a valuable tool for determining the optimal timing of embryo transfer.

Moreover, some studies have reported that a high density of pinopods (>85%) is associated with improved pregnancy rates, whereas their absence or low abundance correlates with reduced implantation potential and an increased risk of spontaneous abortion [31].

Despite these observations, the clinical value of pinopods as reliable biomarkers of endometrial receptivity remains controversial. Several studies have questioned their specificity, reporting their presence across different phases of the luteal cycle, thereby limiting their usefulness as a precise indicator of the WOI [33]. Consequently, although pinopods represent an intriguing morphological marker, their role in clinical practice remains uncertain [34].

#### 2.2.1. Cytokines and Growth Factors

Cytokines and growth factors, which are proteins produced by cells, can interact with specific receptors on cell surfaces, influencing various functions of endometrial cells. These substances play crucial roles in regulating cell proliferation, differentiation, and apoptosis through various mechanisms like autocrine (acting on the same cell), paracrine (acting on nearby cells), or endocrine (acting on distant cells) signaling pathways [6].

Cytokines originating from both the uterine lining and the embryo likely influence the interaction between the mother and embryo, potentially improving the receptivity of the endometrium by regulating the expression of proteins involved in adhesion and anti-adhesion processes. The endometrium is a crucial site for cytokine production and activity, facilitating communication between the embryo and endometrium through shared cytokine receptors. Growth factors and cytokines, which are regulated by hormones, play a crucial role in endometrial development. Endometrial decidualization triggers the production of various growth factors and cytokines that work together to facilitate the decidualization process and support trophoblast invasion. Cytokines not only respond to hormones but also interact with each other in a cascade, contributing to a redundant system [35]. Key cytokines and growth factors believed to contribute to human implantation by enhancing endometrial receptivity notably include leukaemia inhibitory factor (LIF), interleukin-1 (IL-1), heparin-binding epidermal growth factor (HB-EGF), colony-stimulating factor-1 (CSF-1), insulin-like growth factor binding protein-1 (IGFBP-1), and keratinocyte growth factor (KGF) [29].

##### Leukaemia Inhibitory Factor

LIF is a pleiotropic cytokine belonging to the IL-6 family, with multifunctional roles, and plays a crucial role in implantation. Its significant presence in the human endometrium, particularly during the secretory phase, highlights its importance in reproductive physiology [36].

LIF exerts its cellular effects by binding to the LIF receptor (LIFR), a heterodimer composed of two transmembrane proteins: LIFR and gp130. The binding of LIF with LIFR recruits gp130, forming a functional receptor complex and activating downstream signal transduction pathways [37]. This receptor complex facilitates LIF’s complex signaling mechanisms, orchestrating various cellular responses critical for successful implantation and the establishment of pregnancy [28].

LIF is involved in critical events during implantation, such as the conversion of the endometrium to a receptive state, embryo-endometrial interaction, stromal decidualization, trophoblast invasion, blastocyst development, and uterine leukocyte infiltration [37]. During the proliferative phase, levels of LIF protein and *LIF* mRNA are at a minimum. However, during the secretory phase, there is a significant increase in both LIF protein and mRNA expression, particularly in the luminal and glandular epithelium. Although LIFR is present during both proliferative and secretory phases, its expression is limited to the luminal epithelium. This expression is also important for pinopod formation, which is believed to be essential for blastocyst implantation in women [36]. Nevertheless, despite its clear biological relevance, the clinical applicability of LIF remains uncertain. While it has shown some potential as a diagnostic and prognostic marker, its overall performance is limited by moderate sensitivity and specificity, as well as by the lack of standardized methodologies and its context-dependent behavior. For these reasons, LIF cannot currently be considered a reliable standalone biomarker for use in clinical practice [38] (Table 1).

##### The Interleukin-1 System

Studies suggest that the immune system plays a significant role in regulating ovarian function through its interaction with cytokines, which are soluble polypeptides of immunologic origin. This interaction involves a reciprocal relationship between the immune system and the reproductive system. The changes observed in the ovary during ovulation resemble an inflammatory-like reaction, and IL-1 (a well-known mediator of inflammation) is found in both animal and human ovarian follicular fluids. As a result, extensive research has been conducted on IL-1 due to its presence and potential significance in ovarian function [39].

The IL-1 system comprises three closely related polypeptides: IL-1 alpha (IL-1α), IL-1 beta (IL-1β), and IL-1 receptor antagonist (IL-1RA). Both IL-1α and IL-1β exhibit a wide range of biological effects, while IL-1RA inhibits the activities of IL-1. IL-1α and IL-1β share a molecular weight of approximately 17.5 kDa but have distinct amino acid sequences with only 26% homology. Despite this, they bind to the same receptors, which are part of the immunoglobulin (Ig) superfamily, consisting of numerous cell surface and secreted proteins [29].

Research has shown a significant increase in IL-1 receptors during the mid-luteal phase in human endometrium, and elevated levels of embryonic IL-1 in culture media may be linked to embryonic implantation following IVF and embryo transfer [28].

IL-1 plays a significant role in the foeto-maternal interface, where trophoblastic cells and decidualized stromal cells produce this cytokine. Its receptor is found in both endometrial epithelial cells and trophoblasts. IL-1 may act as one of the initial signals from the blastocyst to the endometrium, as it enhances endometrial secretion of prostaglandin E2, LIF, and integrin β3 subunit expression. While IL-1 typically stimulates fibroblast proliferation, it remarkably inhibits the growth of normal human endometrial stromal cells, suggesting a role in endometrial homeostasis. The presence, localization, cycle-dependent changes, and functions of the IL-1 system in the human endometrium suggest a potential autocrine/paracrine/intracrine action relevant for decidualization and implantation [40].

Moreover, human endometrial epithelial cells consistently produce both IL-1α and IL-1RA throughout the menstrual cycle, with mRNA expression for IL-1 receptor type 1 remaining constant. These molecules play crucial roles in regulating interactions between embryos and endometrial cells, influencing the susceptibility of endometrial epithelial cells to apoptosis during implantation. A balanced IL-1α to IL-1RA ratio is essential for the successful implantation of human embryos [29].

Despite these observations, the clinical relevance of the IL-1 system as a biomarker remains uncertain. Although a single case–control study reported very high sensitivity and specificity, these findings are limited by the small sample size and lack of external validation, reducing their generalizability [41] (Table 1). In addition, the absence of standardized sampling procedures and variability across studies has so far prevented the adoption of IL-1–based assays as routine clinical tools [42].

##### Interleukin-6

IL-6, another cytokine, is thought to play a role in trophoblast growth and placental development in humans, particularly during the mid-secretory phase of the menstrual cycle, which corresponds to the WOI [43]. IL-6 expression has been observed to increase during this phase, particularly in glandular and luminal epithelial cells, suggesting its involvement in the implantation process. However, despite its presence, studies comparing IL-6 secretion in infertile and fertile women during the fertile days, typically between LH + 6 and LH + 13, found no significant difference. This suggests that while IL-6 may be involved in early reproductive processes, its role in uterine receptivity during the fertile window might be redundant [44]. During early implantation, there is a notable increase in endometrial pro-inflammatory cytokines, such as IL-6, LIF, and TNF-α, produced by both endometrial cells and immune cells recruited to the implantation site [45]. IL-6 is one of the most studied cytokines in endometriosis and shows moderate diagnostic performance, with sensitivity and specificity generally ranging from 43.9% to 97.7% and 61.1% to 94.4%. However, its clinical utility is limited by significant variability between studies, the lack of standardized cutoff values, and overlap with other inflammatory conditions [46] (Table 1).

Overall, IL-6 can be considered a promising research biomarker; however, it is not yet an established clinical marker for the determination of the WOI.

##### Interleukin-11

Initially known for its role in hematopoiesis and its anti-inflammatory properties, IL-11 has been proposed as a key player in human implantation [47]. Research has revealed the presence of both IL-11 and the IL-11 receptor alpha (IL-11Rα) in the endometrium. While IL-11 is expressed by all major endometrial cell types with fluctuations throughout the menstrual cycle, its highest levels have been detected in decidualized stromal cells during the late stages of the cycle. In contrast, IL-11Rα expression remains constant, suggesting that the timing and pattern of IL-11 expression may be critical for its function in the endometrium [47]. Although it is recognized as a molecular biomarker of endometrial receptivity, the clinic currently relies on broader transcriptomic tests (analyzing hundreds of genes) to define the personalized WOI (Table 1).

##### Interleukin-15

IL-15, a member of the cytokine family, weighs around 14–15 kDa and shares structural similarities with IL-2. Its primary role involves activating neutrophils, macrophages, and T cells. Crucially, it serves as a core chemokine that regulates lymphocyte function and maintenance. IL-15 is crucial for the development of NK cells in the bone marrow and enhances the proliferation, cytokine production, and cytotoxicity of activated blood NK cells. However, unlike its effects on blood NK cells, IL-15 does not convert uNK cells into highly cytolytic cells, a trait vital at the maternal-fetal interface, where cytolytic activity could harm trophoblasts. In the human uterus, IL-15 likely supports the survival and expansion of uNK cells. Both *IL*-15 mRNA and protein are present in non-pregnant human endometrium, decidua, and placenta. The protein is localized peri-vascularly in secretory phase stromal fibroblasts, in glandular epithelial cells during the proliferative phase, and in decidua during the first trimester of pregnancy [48].

When human endometrial stromal fibroblasts undergo decidualization induced by either cyclic adenosine monophosphate (cAMP) or progesterone, an increase in *IL*-15 mRNA expression and protein secretion is observed. This enhancement is further amplified in the presence of interferon-gamma (γ), although interferon-γ alone cannot induce IL-15 production. uNK cells are likely a source of interferon-γ in the endometrium, suggesting the possibility of increased IL-15 production from decidualizing cells due to the presence of adjacent uNK cells. On the other hand, IL-1β appears to play a contrasting role, acting as a negative regulator of *IL*-15 mRNA and protein expression during in vitro decidualization [49].

IL-15 monitoring shows clinical promise for tailoring therapies in patients with recurrent implantation failure but has not yet entered large-scale routine diagnostic protocols [50] (Table 1).

##### Epidermal Growth Factor Family of Growth Factors & Heparin Binding-Epidermal Growth Factor

The EGF family of growth factors, which includes epidermal growth factor (EGF), TGF-α, heparin binding-EGF (HB-EGF), amphiregulin, β-cellulin, epiregulin, and neuregulins, is believed to play roles in various developmental, physiological, and pathological processes. These factors interact with receptor subtypes from the *erythroblastic oncogene B* (*ErbB*) gene family—namely *ErbB1* (EGF-R), *ErbB2*, *ErbB3*, and *ErbB4*—which share structural features but differ in ligand specificity and kinase activity [51].

EGF has been identified in the human endometrium throughout the menstrual cycle, in gestational decidua, and in first-, second, and third-trimester placenta. During the proliferative phase of the endometrium, moderate levels of EGF are found in stromal cell cytoplasm, with intense localization during the secretory phase. EGF is also detected in decidual and trophoblastic cells. Previous research indicates that EGF is crucial for trophoblast invasion, differentiation, and proliferation, suggesting its significant role in various implantation processes [49]. However, despite its biological relevance, EGF alone is not used in clinical practice to assess endometrial receptivity. Instead, clinical evaluation generally relies on broader transcriptomic platforms, such as the endometrial receptivity array (ERA), which analyzes the expression of hundreds of genes (Table 1).

*HB-EGF*, which shares a receptor with EGF and TGF-α, is expressed in both endometrial stromal and epithelial cells. It has been demonstrated to regulate various processes, including endometrial cell proliferation, glandular epithelial secretion, and decidualization. Its expression peaks during the mid-secretory phase, suggesting its role in regulating blastocyst implantation [51]. In humans, *HB-EGF* plays a crucial role in implantation and embryonic development, with its expression peaking during the receptive period. Studies indicate that *HB-EGF* enhances blastocyst hatching and facilitates the adhesion of endometrial epithelial cells to the trophectoderm of the blastocyst. Thus, *HB-EGF* emerges as a potent growth factor for the blastocyst, playing a significant role in preimplantation embryonic development and implantation across various species through paracrine mechanisms [52]. *HB-EGF* analysis is often used in research settings or specific studies on endometrial quality, rather than as a standard and routine diagnostic test in all IVF centers (Table 1).

##### Colony Stimulating Factor-1

CSF-1, a homodimeric glycoprotein initially recognized as a growth factor for mononuclear phagocytic cells, significantly increases in the human endometrium during the first trimester of pregnancy compared to non-pregnant levels. This elevation is attributed to uterine synthesis of CSF-1, as evidenced by the presence of *CSF-1* mRNA throughout gestation, with the highest expression observed during the first trimester. Consequently, CSF-1 likely plays an important role in both ovulation and early pregnancy [53].

Despite its biological role, its clinical utility as a reliable marker is not yet established. Studies indicate that current evidence often comes from small patient groups and lacks prospective, controlled studies to validate its definitive diagnostic use [54] (Table 1).

##### Transforming Growth Factor Beta

The TGF-β family, consisting of three isoforms, plays a crucial role in tissue development and growth. Their presence at the maternal-fetal interface, along with their effects on cell proliferation, differentiation, and migration, suggests their involvement in implantation processes [55]. In the human endometrium, TGF-β isoforms are localized in stromal, epithelial, and decidual cells, with TGF-β2 being more prevalent in stromal cells and TGF-β1 and TGF-β3 showing equal intensity. TGF-β1 promotes trophoblast attachment to the endometrium during implantation by stimulating extracellular matrix (ECM) production [56].

During early pregnancy, TGF-β regulate maternal immunotolerance and various implantation-related molecules, such as vascular endothelial growth factor (VEGF), matrix metalloproteinase-9 (MMP-9), IGFBP-1, and LIF. However, they also inhibit trophoblast invasion by modulating the secretion of enzymes. Elevated TGF-β levels in preeclampsia may impair implantation. Thus, TGF-β serves as a signaling molecule in the endometrium, influencing trophoblast invasion during implantation and placentation [57]. Its role in decidualization remains unclear, although other members of the TGF-β superfamily, such as macrophage inhibitory cytokine-1 (MIC-1), may contribute to this process by promoting decidualization [58]. Furthermore, seminal plasma TGF-β is adsorbed onto the sperm surface, demonstrating that sperm transport is an additional mechanism by which seminal plasma factors can cross the cervix to access the endometrium [59].

It is also important to underline that the postcoital inflammatory response in women is primarily mediated by cytokines belonging to the TGF-β family. Specifically, TGF-β induces the expression of female proinflammatory and immunomodulatory factors, including colony-stimulating factor-2 (CSF-2) and IL6, molecules involved in promoting endometrial receptivity and, consequently, in preparing for pregnancy. Furthermore, TGF-β exerts an important immune diversion function, promoting maternal immunological tolerance through the induction and expansion of regulatory T cells [60].

In summary, members of the TGF-β family represent potential molecular biomarkers to assess endometrial receptivity, especially in cases of unexplained infertility or ART failures, even if their measurement is not yet the single and universal “gold standard” in daily clinical practice compared to more extensive gene panels [61] (Table 1).

##### Insulin-like Growth Factor Binding Protein-1 and Insulin-like Growth Factor-1

The insulin-like growth factor (IGF) family and IGFBPs play vital roles in various processes within the endometrium, including growth, differentiation, angiogenesis, and apoptosis. IGFBP-1 is secreted approximately 10 days after the LH peak, aligning with the closure of the WOI. This timing suggests its involvement in cellular proliferation and differentiation, which are crucial for decidualization and maintaining pregnancy [62]. As one of six homologous proteins, IGFBP-1 regulates the mitogenic and metabolic effects of IGFs (IGF-1 and IGF-2), which are essential for growth, apoptosis, metabolism, and development. It is predominantly expressed in the liver, kidney, decidualized endometrium, and luteinizing granulosa cells. In the endometrium, IGFBP-1 protein and mRNA are localized to pre-decidual stromal cells in the late secretory phase and to decidual cells during pregnancy. It enhances gelatinolytic activity and inhibits trophoblast migration into decidualized stromal multilayers [45].

IGFBP-1 has been suggested as the primary IGFBP synthesized by decidual cells, interacting with trophoblast-synthesized IGF-2 to facilitate cell-to-cell communication between trophoblasts and the decidua, regulating invasion [63]. While IGF-2 and IL-1β inhibit IGFBP-1, IL-1β also inhibits decidualization. In contrast, other growth factors, such as TGF-β, stem cell factor (SCF), CSF-1, and LIF, do not impact IGFBP-1 expression [64]. The specific evaluation of the IGF family (such as IGFBP-1) is usually used in research studies or to evaluate the correct functional maturation of the endometrium, especially in cases of recurrent miscarriages or repeated IVF failures, but not in the diagnostic field (Table 1).

##### Vascular Endothelial Growth Factors

VEGF is a potent stimulator of endothelial cell growth in laboratory settings and plays a key role in vasculogenesis and embryogenesis across various in vivo models [65]. Apart from VEGF-A, this growth factor family includes placental growth factor (PlGF), VEGF-B, VEGF-C, and VEGF-D. VEGF binds directly to two types of surface receptors: VEGFR-1 (Flt-1) and VEGFR-2 (Flk-1/KDR), which are classified as type 3 receptor tyrosine kinases [66]. VEGFR-1 also interacts with PlGF, a protein closely related to VEGF, and is primarily expressed in specific tissues, such as the placenta. In humans, the expression of *VEGF-A* varies across the menstrual cycle. Specifically, VEGF189, a variant of VEGF-A bound to the extracellular matrix, is dependent on progesterone levels in the uterus. Both *VEGF* mRNA and protein are detectable in endometrial stromal, glandular, and endothelial cells during the mid-secretory phase, with the highest levels observed during the mid-to-late proliferative phase. During this period, both VEGFR1 and VEGFR2 are present in microvessels, suggesting a correlation between increased microvascular density and vascular permeability. Furthermore, the secretion of VEGF into the lumen of endometrial glands in human cell cultures suggests that apically secreted VEGF may play a role in signaling processes related to blastocyst development and implantation within the endometrium [45]. It is recognized in clinical research as an important molecular biomarker of endometrial receptivity, although its use in daily clinical practice is often associated with other ultrasound or molecular parameters rather than as a single, isolated test [67] (Table 1).

##### Granulocyte Colony Stimulating Factor (G-CSF)

Granulocyte colony stimulating factor (G-CSF) is a member of the CSF family. It has been detected in the ovary, primarily in granulosa cells of the follicles and luteal cells, as indicated by Western blotting and immunohistochemistry. Higher levels of G-CSF have been observed in follicular fluid compared to serum during ovulation. Elevated serum levels of G-CSF have also been found during implantation in successful natural cycles, as well as following successful IVF or intracytoplasmic sperm injection (ICSI) attempts. This suggests that G-CSF may play a role in the implantation process. Additionally, the presence of G-CSF in follicular fluid could serve as a marker for assessing individual oocyte immune tolerance, particularly in women with low ovarian reserve, and help in determining the most suitable ovarian stimulation protocol [68].

##### Keratinocyte Growth Factor

Keratinocyte growth factor (KGF), also known as fibroblast growth factor-7 (FGF-7), belongs to the fibroblast growth factor (FGF) family and was initially sourced from human embryonic lung fibroblasts [69]. KGF plays a crucial role in the development of various organs, including the lungs, skin, mammary glands, digestive tract, and reproductive tracts. It primarily acts in a paracrine manner, targeting epithelial cells by binding to the KGF receptor (KGFR or FGFR-2IIIb), which is mainly localized in epithelial cells [70]. Both KGF and KGFR are expressed in various cancer tissues, where their effects on cell behaviours such as growth, motility, invasion, and differentiation depend on the organ and tissue type. In cancers, KGF is implicated in stimulating the motility of estrogen receptor-positive (ER-positive) breast cancer cells and inhibiting apoptosis. It also downregulates the expression of ER and progesterone receptor (PR), contributing to increased resistance to tamoxifen therapy [69].

In the human endometrium, *KGF* expression rises during the luteal phase of the menstrual cycle, while *KGFR* mRNA expression is influenced by estrogen. Both *KGF* and *KGFR* transcripts are detected in endometrial cancer cells, with KGF administration stimulating cancer cell proliferation [71].

##### Human Leukocyte Antigen-G

HLA-G, a non-classical HLA class I molecule with immunoregulatory function, may represent a relevant biomarker of endometrial receptivity and maternal-foetal tolerance. Larsen et al. [72] demonstrated the presence of the soluble form of HLA-G (sHLA-G) in human seminal plasma and HLA-G expression in the testis and epididymis, while it was not observed in seminal vesicles. Weak expression was also detected in hyperplastic prostatic tissue. The authors therefore hypothesized that HLA-G contributes to the immune modulation of the male reproductive tract and that its presence in seminal plasma may participate in the immunological priming of the female uterine environment prior to implantation. Subsequently, Schallmoser et al. [73] quantified sHLA-G levels in 106 seminal plasma samples from men undergoing ART, confirming the presence of sHLA-G also in testicular biopsy samples. In this study, sHLA-G levels did not differ significantly between normozoospermic men and men with reduced seminal quality, nor were they significantly associated with the partner’s pregnancy outcome. However, the authors suggested that elevated sHLA-G concentrations may contribute, together with other immunomodulatory factors, to the establishment and maintenance of pregnancy through long-term modulation of the uterine microenvironment. Furthermore, a negative correlation was observed between male age and total sHLA-G concentration in seminal plasma (Table 1).

**Table 1:** Overview of key endometrial receptivity biomarkers, including their reported clinical indications, diagnostic performance (sensitivity and specificity where available), and current validation status.

Biomarkers	Clinical Indication	Sensitivity	Specificity	Validation Status	Reference
Leukaemia Inhibitory Factor (LIF)	endometrial receptivity	86.7%	100%	limited due to lack of standardization	[38]
The Interleukin-1 System	regulating ovarian function in human ovarian follicular fluids	100%	100%	need to standardize sampling techniques (biopsy or aspiration)	[41]
Interleukin-6 (IL-6)	Advanced Endometriosis	43.9–97.7%	61.1–94.4%	not used in clinical diagnosis	[46]
Interleukin-11 (IL-11)	human implantation and determination of WOI	unspecified	unspecified	not used in clinical diagnosis	[47]
Interleukin-15 (IL-15)	implantation failure	85.0%	68.4%	not used in clinical diagnosis	[50]
EGF	Edometrial receptivity	unspecified	unspecified	not used in clinical diagnosis	[49]
Colony-Stimulating Factor-1 (CSF-1)	growth factor for mononuclear phagocytic cells	unspecified	unspecified	not used in clinical diagnosis	[54]
Transforming Growth Factor Beta (TGF-β)	implantation processes	unspecified	unspecified	not used in clinical diagnosis	[55]
Insulin-like growth factor (IGF)	various processes within the endometrium, including growth, differentiation, angiogenesis, and apoptosis	unspecified	unspecified	not used in clinical diagnosis	[62]
Vascular Endothelial Growth Factors (VEGF)	angiogenic factor	79.08%	52.04%	clinical research	[67]
Granulocyte Colony Stimulating Factor (G-CSF)	implantation successful	unspecified	unspecified	not used in clinical diagnosis	[68]
Keratinocyte growth factor (KGF)	in the development of various organs	unspecified	unspecified	not used in clinical diagnosis	[69]
HLA-G	Endometrial receptivity	unspecified	unspecified	not used in clinical diagnosis	[72,73]

##### Cell Adhesion Molecules

During WOI, both the human endometrium and the embryo expressed adhesion molecules crucial for blastocyst attachment to the uterine mucosa, including mucins and integrins. Integrins, identified by Lessey et al. in the 1990s [74], are significant biomarkers of endometrial receptivity. Specific integrin subunits, such as α1, α4, β1, β3, and β5, show varying expression levels throughout the menstrual cycle [59]. β3 integrin is particularly indicative of endometrial receptivity during the secretory phase [75]. The expression of αVβ3 integrin at the foeto-maternal interface in several species, including sheeps, pigs, baboons, and humans, during blastocyst attachment and implantation, supporting its role in the implantation process [75].

uNK cells, regulated by progesterone-induced IL-15 secretion, play a crucial role in endometrial receptivity during the mid-luteal phase. They are distinct from circulating NK cells and are identified by CD56bright, CD16dim surface antigen expression [69]. uNK cells promote decidualization and materno-foetal tolerance through interactions with trophoblasts and remodeling of endometrial vasculature [69]. Abnormal uNK cell levels are associated with RIF and miscarriage. However, solely measuring uNK cell count may not reliably predict pregnancy outcomes, emphasizing the need for standardized assessment methods [76] (Table 2).

##### Novel Biomarkers for Assessing Endometrial Receptivity

Recent advancements in endometrial research have identified novel biomarkers crucial for fertility and reproductive health, including the microbiome, extracellular vesicles (EVs), microRNAs (miRNAs), and glycobiology, representing promising avenues for further research and potential clinical applications in fertility assessment and management. Similar to the gut microbiome, the reproductive tract microbiome plays a significant role in fertility [82]. A dominant presence of *Lactobacillus* spp. in the vaginal microbiome, particularly during the mid-luteal phase or embryo transfer, correlates with improved fertility outcomes, while higher microbial diversity and reduced *Lactobacillus* spp. Dominance is associated with infertility, RIF, and recurrent miscarriage [83]. Although the endometrial microbiome shares similarities with the vaginal one, there is ongoing debate about the true prevalence of *Lactobacillus* spp. within it [83]. A Dutch research team developed a diagnostic test using mid-luteal vaginal microbiome swabs to predict endometrial receptivity based on *Lactobacillus* spp. dominance, with favourable microbiome profiles correlating with higher pregnancy rates [76]. Despite the availability of clinical tests, it remains unclear whether microbiome imbalances cause fertility issues or merely reflect underlying dysfunctions, with hormonal factors such as 17β-estradiol potentially influencing the vaginal microbiome and impacting fertility outcomes [84]. Another hypothesis highlights the endometrium as a perceptive biosensor capable of identifying embryos with low developmental potential, while embryos may communicate endometrial readiness through molecules enclosed in lipid-soluble structures known as EVs [85]. Embryo-derived EVs in spent culture media may indicate embryo competence, while endometrial-derived EVs (eEVs), which peak during the mid-luteal phase, support implantation by promoting trophoblast invasion. These EVs also carry miRNAs that regulate gene expression in embryonic or endometrial cells. Specific miRNAs have been linked to receptivity; for instance, mi-Let-7-a enhances receptivity by increasing β-catenin expression, whereas miR-30b in endometrial epithelial cells impairs implantation [86]. In parallel, early-stage glycobiology research has identified molecules potentially relevant to implantation. For example, increased placental expression of galectin-7 during week 6 of pregnancy, particularly at sites of trophoblast invasion, has been linked to miscarriage, likely by impairing the endometrium’s ability to select viable embryos, consistent with the biosensor hypothesis [87]. Glycans and their mediators also emerge as potential biomarkers during early embryo adhesion and invasion. In human cells, progesterone stimulates a specific glycosyltransferase that is involved in glycosylating embryonic adhesion molecules, thereby supporting trophoblast invasion. This enzyme is notably deficient in trophoblast cells from patients with miscarriages who have poorly developed decidual vasculature [80].

##### Other Possible Biomarkers

**Figure 2:**
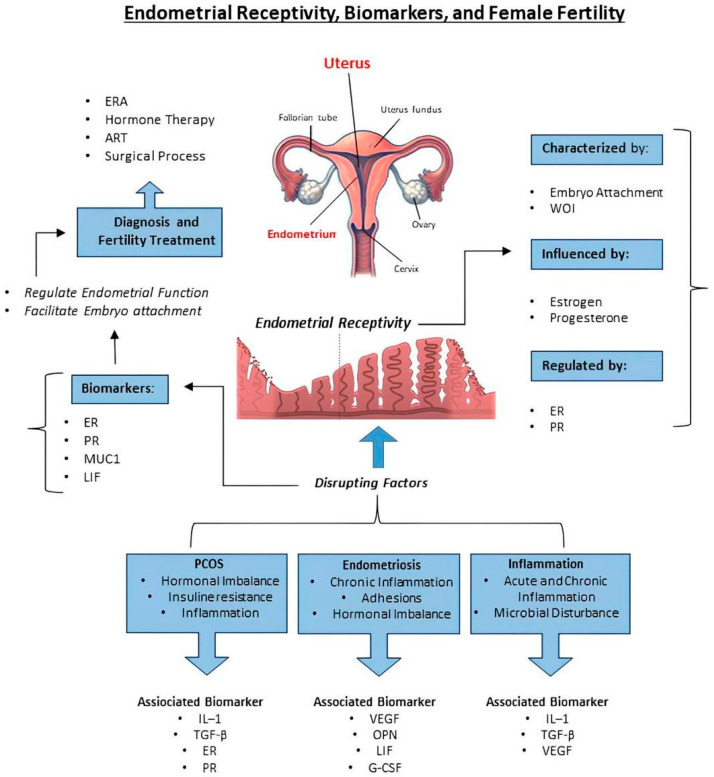
Schematic representation of endometrial receptivity and its regulation. The intricate interplay between hormonal regulation (estrogen, progesterone), disrupting factors (polycystic ovary syndrome—PCOS, endometriosis, inflammation each with associated biomarkers), and biomarkers governing endometrial receptivity is illustrated. Biomarkers facilitate embryo attachment and diagnosis, guiding fertility treatments such as endometrial receptivity array (ERA), hormone therapy, assisted reproductive technology (ART), and surgical interventions.

Calcitonin, a peptide hormone produced by the parafollicular cells of the thyroid gland in response to elevated serum calcium levels, also appears in various tissues, including the uterus. Studies have demonstrated calcitonin expression in the human endometrium during the implantation phase, with calcitonin mRNA peaking in the uterine lining during Days 19–21 of the menstrual cycle, aligning with the WOI. Progesterone has been shown to induce calcitonin gene expression in the endometrium [80]. Another class of potential biomarkers is the *HOX* genes, which encode transcription factors essential for embryonic development and endometrial gene regulation across menstrual cycles [88]. Organized into four clusters (A, B, C, D) across separate chromosomes, these genes produce transcription factors that modulate downstream gene targets and are characterized by a conserved 60-amino acid DNA-binding domain [89]. Among them, *HOXA10* and *HOXA11* play a key role in implantation, exhibiting increased expression in the endometrial glands and stroma during the mid-secretory phase, which persists into pregnancy. This upregulation is hormonally driven, as demonstrated by Taylor et al., who reported that ovarian hormones regulate the expression of *HOXA10* and *HOXA11* in the human endometrium. Peak expression of these genes coincides with endometrial receptivity, marking them as crucial players in implantation [90]. Osteopontin (OPN), also known as secreted phosphoprotein 1, is another important biomarker candidate. This multifunctional ECM protein/cytokine, varying from 25–75 kDa due to modifications like phosphorylation and glycosylation, mediates cell adhesion, migration, and signaling through interactions with cell surface receptors [75]. OPN contributes to implantation by forming part of the histotroph, a uterine secretion essential for conceptus adhesion and signaling at the uterine-placental interface [91]. It is also expressed in the uterine stroma during decidualization and in immune cells of the uterus and placenta, where it regulates immune behavior and cytokine production [91]. As a major component of histotroph during early pregnancy, OPN supports uterine gland secretions and helps maintain pregnancy during the first trimester in humans [75]. A schematic representation of endometrial receptivity and the biomarkers that play a crucial role in facilitating embryo attachment and guiding fertility treatments is shown in Figure 2.

##### Semen-Induced Immunological Tolerance as a Functional Biomarker of Endometrial Receptivity

Over the past two decades, it has become clear that bioactive agents present in the noncellular fraction of seminal fluid play a key role in modulating the female immune response and, consequently, in defining endometrial receptivity. After intercourse, the cervix and, potentially, the endometrium and fallopian tubes respond to seminal signals through profound changes in gene expression. Specifically, seminal fluid interacts with the endometrial luminal epithelium, inducing or repressing hundreds of mRNAs and numerous microRNAs, many of which are involved in regulating the immune response. These changes drive the local synthesis of cytokines and chemokines that are released into the stromal and luminal compartments, promoting the recruitment of macrophages, dendritic cells, and granulocytes into the endometrial stroma within hours [59]. Neutrophils and macrophages contribute to the removal of cellular debris and microorganisms introduced during intercourse, as well as participating in the selection of the most competent sperm. Dendritic cells, on the other hand, play a crucial role in inducing immunological tolerance: they internalize antigens present in seminal fluid, migrate to the draining lymph nodes, and, through cross-presentation, induce the expansion of regulatory T cells (Treg) specific for paternal antigens, including major histocompatibility complex antigens [59]. The generated Tregs subsequently return to the endometrium, where they promote an immunological condition permissive for embryonic implantation. Since the embryo expresses the same paternal antigens previously encountered in seminal fluid, the presence of a stable and functionally competent population of Tregs is an essential prerequisite for maintaining pregnancy. These cells support trophoblastic invasion and the vascular remodeling necessary for placental development, simultaneously suppressing inflammatory responses that could compromise the survival of the semi-allogeneic embryo. Treg generation depends largely on factors present in seminal plasma, particularly TGF-β, which is produced latently by the seminal vesicles and activated in the female genital tract after ejaculation [59]. TGF-β is therefore one of the main mediators of immune tolerance associated with endometrial receptivity and, together with the cytokine, chemokine, and microRNA profiles induced by seminal fluid, could be a promising functional biomarker of the endometrium’s ability to support implantation. The importance of this immunological priming is further supported by clinical studies in assisted reproduction, which show improved implantation and pregnancy rates with exposure to the partner’s semen. Although intercourse is not essential in IVF cycles, the absence of seminal signals could contribute to reduced endometrial receptivity, suggesting that the evaluation of seminal fluid-induced immune tolerance mechanisms should be considered among the potential biological indicators of a receptive endometrium [59].

## 3. Advances in Diagnosis and Treatment

### 3.1. Commercially Available Approaches to Evaluate Endometrial Receptivity

The diagnosis of endometrial receptivity has its own share of significant challenges, and the majority of the available tests have been subjective and lack accuracy and predictive value. Several approaches are available for evaluating endometrial receptivity in clinical settings. These approaches aim to assess various molecular, histological, and physiological markers associated with endometrial receptivity to predict the optimal timing of embryo transfer in ART such as IVF. Some of the commonly used commercially available methods include the following.

#### 3.1.1. Endometrial Receptivity Array

In 2009, Haouzi and colleagues [92] identified an exclusive transcriptomic signature in human endometrial biopsies, focusing on five genes (*laminin subunit beta 3—LAMB3*, *microfibril-associated protein 5—MFAP5*, *angiopoietin-like protein 1—ANGPTL1*, *prokineticin 1—PROK1*, *nuclear factor erythroid 2-related factor 2—NRF2*) modulated during the WOI. These genes are overexpressed in the mid-secretory phase and are involved in ECM remodeling, angiogenesis, and endothelial microenvironment formation. Additionally, eleven genes have been associated with a receptive endometrial state. These findings led to the development of the WIN-TEST, the first commercially available predictive tool for assessing endometrial receptivity [93].

The analysis of the transcriptomic signature of the WOI using microarray technology has led to the development of a molecular diagnostic method, namely the ERA, which serves as a valuable tool in pinpointing the precise window when the endometrium is most receptive to embryo implantation [80]. It classifies endometrial samples as pre-receptive, early receptive, receptive, late receptive, or post-receptive. This receptivity is facilitated by the maturation of the endometrium, largely driven by hormonal fluctuations during menstruation [94].

The ERA test identified 238 genes with differential expression during the transition of the endometrial state from the pre-receptive to the receptive state. Using artificial intelligence, the test accurately determines the WOI for each patient by comparing the genetic profiles of samples taken on specific days of the cycle. Although some studies have shown a slight decrease in its sensitivity and specificity, the test remains more accurate than traditional histological methods, allowing for the customization of frozen embryo transfer (FET) plans, adapting to both hormone replacement therapy and natural cycles, and improving IVF outcomes 145 [95].

However, new data from studies on serum samples collected from patients who underwent the ERA test are allowing the identification of new biomarkers that appear to be very reliable, reassessing the potential of the ERA test in predicting the WOI. Some miRNAs, such as miR-31, let-7a, miR-182-5p, and miR-135a-5p, have emerged as promising biomarkers. In particular, miR-31 has been identified as a biomarker for monitoring endometrial receptivity and predicting implantation failure. Additionally, a recent study identified tsRNAs (small RNAs derived from tRNA) as non-invasive biomarkers for endometrial receptivity, suggesting that they could be used in the future to prevent implantation failure or treat infertility related to receptivity.

#### 3.1.2. BeREady Test

The BeREady test is a commercialized predictive tool that utilizes an innovative method called targeted allele counting by sequencing (TAC-seq) to assess the expression of 67 biomarker genes indicative of endometrial receptivity [79]. It categorizes endometrial samples into different phases of receptivity, including pre-receptive, early receptive, receptive, late receptive, or post-receptive. Clinicians conduct endometrial biopsies using a pipelle in affiliated clinics, and the samples are then dispatched for assessment and biomarker analysis. The test results are available within 14 days. Despite its reliance on validated biomarkers identified in previous research, there is currently a lack of studies evaluating the BeREady test’s accuracy in predicting the WOI or its impact on clinical outcomes [96]. While the ERA test analyzes the expression of 238 genes involved in endometrial receptivity using microarray technology, the BeREADY model adopts a different approach to biomarker selection. The genes included in the BeREADY panel are derived from a broad meta-analysis of biomarkers associated with endometrial receptivity, subsequently integrated with eleven additional genes deemed relevant for identifying the WOI and four housekeeping genes used as internal references for data normalization. The ERA test was developed to recognize the typical transcriptomic profile of the receptive endometrium by comparing the patient’s sample with the genetic pattern observed during the receptive phase of the cycle. The platform used is based on a customized microarray containing 238 genes differentially expressed between pre-receptive and receptive endometrium. BeREADY, on the other hand, uses a TAC-seq methodology, developed to reduce the costs and limitations of global transcriptomic profiling. The test’s commercial panel includes 67 overall biomarker genes for endometrial receptivity. Another distinctive feature of BeREADY is its use of unique molecular identifiers (UMI) technology. By adding unique molecular markers to each transcript before amplification, the system estimates the original number of RNA molecules present in the sample. This limits the biases introduced by PCR amplification and achieves more accurate and reliable gene expression quantification [13,97].

#### 3.1.3. Window Implantation Test

The WIN-Test was the first commercial test developed to assess endometrial receptivity and can more accurately identify the WOI. The test is based on reverse transcription-quantitative polymerase chain reaction (RT-qPCR) quantification of the expression of 11 endometrial genes selected because they are significantly more expressed during the receptive phase than during the pre-receptive phase of the endometrium. The genes included in the panel are *MFAP5*, *ANGPTL1*, *PROK1*, *NRF2*, *LAMB3*, *BCL2L10*, *CD68*, *TRPC4*, *SORCS1*, *FST*, and *KRT80* [13].

These biomarkers were selected by comparing endometrial biopsies obtained during the pre-receptive (LH+2) and receptive (LH+7) phases. Specifically, the genes were chosen based on an absolute fold change greater than 2 and a false discovery rate less than 0.05. The first validated genes—*LAMB3*, *MFAP5*, *ANGPTL1*, *PROK1*, and *NRF2*—are implicated in ECM remodeling, angiogenesis, and the formation of endothelial fenestrations—processes considered essential for embryo implantation [13].

To perform the test, an endometrial biopsy is performed between LH+6 and LH+9 in natural cycles, or between P+5 and P+9 in progesterone-substituted cycles. The result is obtained within approximately 5 days of receiving the sample. The endometrium is defined as “receptive” when the average expression of the 11 genes reaches at least 70% of the positive control; values between 50% and 70% identify a “partially receptive” state, while values below 50% are classified as “non-receptive” [13].

A prospective study of 217 women with RIF showed that the duration of the WOI varies considerably from patient to patient: in 78.5% of cases, the receptive window lasted less than 48 h, while in a minority of patients it was shorter than 24 h or longer than 48 h [98].

### 3.2. Therapeutic Interventions and Approaches

Therapeutic approaches to enhance endometrial receptivity involve surgical interventions and targeted immune cell therapies, crucial for successful embryo implantation and pregnancy. The first approach improves endometrial receptivity and enhances the chances of successful implantation and pregnancy in patients undergoing ART by creating controlled injury to the endometrium, which stimulates a healing response that may increase receptivity. The second approach aims to regulate the immune environment within the uterus by modulating the activity of specific cell types, promoting immune tolerance, and optimizing tissue remodeling. These approaches focus on balancing cytokine levels, promoting growth factor expression, and enhancing progesterone production to create a favourable environment for embryo implantation. These strategies offer promise for addressing RIF and improving pregnancy outcomes, but further clinical validation is necessary.

#### 3.2.1. Endometrial Injury (Scratching)

Endometrial injury, often performed through endometrial scratching using a pipelle, has been proposed as a method to modulate endometrial receptivity. This process intentionally damages the endometrium, stimulating a healing response that has been hypothesized to promote decidualization, the recruitment of immune cells (such as uNK cells, cytokines, and growth factors), and angiogenesis [99].

Mechanical trauma may also delay endometrial maturation, enhancing synchronization between the endometrium and the embryo, and promoting the expression of genes beneficial for implantation. Some studies, including those by Gibreel and Parsanezhad, have reported improved pregnancy outcomes in IVF/ICSI and intrauterine insemination (IUI) cycles; however, these findings remain inconsistent and are not supported by robust evidence of improved live birth rates [100]. Overall, despite its biological plausibility and ongoing clinical interest, endometrial scratching has not demonstrated clear clinical benefit in terms of live birth outcomes and therefore cannot be recommended as a routine intervention in assisted reproduction.

#### 3.2.2. Immunomodulation

Current evidence indicates that successful embryo implantation depends not only on the presence of molecular markers of endometrial receptivity, but also on finely tuned immune responses that promote maternal–foetal tolerance and prevent embryo rejection. During decidualization, leukocytes are recruited into the uterus and, in early pregnancy, represent approximately 40–50% of decidual cells. Of these, approximately 70% belong to the innate lymphoid cell compartment, predominantly uNK cells, which in the decidua assume a different phenotype than systemic NK cells and support implantation and placentation rather than exerting cytotoxic activity.

Therapeutic approaches to enhance endometrial receptivity involve regulating uNK cell activity with cytokines and inhibitory receptor blockades, modulating dendritic cells’ function through cytokine regulation, regulating maternal T-cell responses, and influencing macrophage activity using pregnancy hormones or intrauterine PBMC administration. These strategies aim to optimize immune cell function and create a favourable immune environment for successful implantation and pregnancy, though further clinical studies are needed to validate their effectiveness [101].

In patients experiencing RIF, intrauterine administration of peripheral blood mononuclear cells (PBMCs) aims to enhance endometrial receptivity by balancing the T-helper type 1 (Th1)/T-helper type 2 (Th2) cytokine ratio and promoting the expression of growth factors, which trigger various cytokine cascades and actions of matrix metalloproteinases. Elevated Th1/Th2 ratios in peripheral blood have been linked to impaired embryo implantation, making immunotherapy with PBMCs a promising approach to address this issue. Additionally, this therapy has been shown to enhance progesterone production in cultured human granulosa luteal cells, crucial for the immunoregulation of embryo implantation [101].

Ovarian steroids, such as progesterone and β-human chorionic gonadotropin (β-hCG), play vital roles in the immunomodulation of embryo implantation. They influence immune cells by binding to their respective receptors, thereby affecting immune tolerance mechanisms that are crucial for successful implantation. β-hCG, specifically, downregulates pro-inflammatory immune responses during pregnancy and increases FS-7-associated surface antigen (Fas) ligand expression in endometrial cells, facilitating trophoblast invasion during the WOI. Furthermore, high levels of peripheral blood regulatory T cells (Treg) have been associated with increased pregnancy rates in IVF treatments, indicating their positive influence on embryo implantation [102].

In this context, HLA-G emerges as one of the main mediators of local immune tolerance. Unlike classical HLA molecules, the non-classical HLA molecules HLA-G and HLA-E expressed by embryonic cells compensate for antigenic discrepancies between mother and embryo and inhibit the cytolytic activity of decidual immune cells. Specifically, HLA-G expressed by extravillous trophoblastic cells interacts with the inhibitory receptors immunoglobulin-like transcript 2 (ILT2), ILT4, and killer cell immunoglobulin-like receptor 2DL4 (KIR2DL4) present on uNK cells, macrophages, and T cells, suppressing cytotoxic responses, promoting the differentiation of regulatory T cells, and contributing to vascular remodeling and placentation. Furthermore, HLA-G appears to act closely with TGF-β, another central mediator of immune tolerance [103].

Another point of interest is the soluble form of HLA-G present in seminal plasma. sHLA-G may constitute an early paternal immunological signal, capable of modulating the immune microenvironment of the female reproductive tract even before implantation. Exposure to seminal plasma during intercourse may induce tolerogenic “preconditioning” to paternal antigens, facilitating subsequent recognition of trophoblastic HLA-G in the early stages of pregnancy. Consistent with this hypothesis, in vitro studies have demonstrated that seminal plasma induces profound transcriptomic changes in human endometrial epithelial and stromal cells, promoting decidualization and stimulating the production of pro-inflammatory and pro-implantation cytokines, including IL-1β, IL-6, and LIF.

Although a meta-analysis reported a significant improvement in clinical pregnancy rates following seminal plasma treatment, no statistically significant increase in live birth rates was observed. Therefore, despite growing biological support for the role of immune mediators and seminal plasma in endometrial receptivity, their clinical application remains limited by the low quality of available evidence, due to the small number of studies, small sample sizes, methodological heterogeneity, and variability of interventions [104].

#### 3.2.3. Stem Cells and Endometrial Regeneration

Endometrial stem cells are crucial for treating uterine lesions and infertility resulting from endometrial damage, due to their ability to differentiate, perform paracrine activity, and exhibit immunomodulatory effects.

Stem cell treatment promotes endometrial regeneration mainly through three mechanisms: differentiation capacity, paracrine activity, and immunomodulatory effects. The differentiation capacity allows mesenchymal stem cells (MSCs) or endometrial stem cells to stimulate endometrial regeneration in patients with intrauterine adhesions by differentiating into endometrial cells [105]. Stem cells not only differentiate into endometrial, stromal and vascular endothelial cells, but also modulate immune cell behaviour through cytokine secretion, thus facilitating endometrial repair. These pluripotent cells exert immunomodulatory effects, regulating the immune response within the damaged endometrium. They are able to modulate the activity of immune cells such as T lymphocytes and macrophages, promoting an anti-inflammatory environment that reduces inflammation and facilitates tissue healing, thus supporting endometrial regeneration [106].

## 4. Conclusions

In conclusion, understanding endometrial receptivity and its biomarkers is crucial in reproductive medicine, as assessing the receptivity of the endometrium can guide personalized embryo transfer strategies to address infertility and pregnancy complications. The evaluation of various biomarkers associated with endometrial receptivity reveals that each plays a crucial role in supporting the processes essential for embryo implantation and communication between the embryo and the endometrium. However, the clinical applicability of each biomarker varies depending on its specificity, sensitivity, and the context in which it is applied.

Despite decades of progress in the characterization of endometrial receptivity, current evidence clearly indicates that no single biomarker, nor any isolated panel, has sufficient accuracy, reproducibility, or clinical robustness to be reliably implemented in routine reproductive medicine. Although molecules such as LIF, integrins, and selected *HOX* genes represent the most biologically consistent markers of receptivity, their predictive performance in clinical settings remains limited and highly context-dependent. Similarly, cytokines and growth factors, including IL-6, IL-15, VEGF, TGF-β, and CSF-1 predominantly reflect inflammatory, vascular, or immune activity rather than specific determinants of the implantation window, resulting in significant overlap between fertile and non-fertile states.

Emerging biomarkers such as extracellular vesicles, endometrial microbiome signatures, and non-coding RNAs offer promising mechanistic insights but remain at an early translational stage, with insufficient standardization and validation to support clinical use. At present, even commercially available transcriptomic platforms, while technologically advanced, are constrained by heterogeneity in study design, limited prospective validation, and uncertain impact on live birth outcomes.

Although therapeutic approaches like endometrial injury, immunomodulation, and stem cell therapies have shown potential in improving endometrial receptivity, they still lack strong evidence. Moreover, advancements in stem cell therapy, particularly the use of MSCs for endometrial regeneration, hold promise in addressing endometrial damage and infertility.

Further clinical validation is needed to confirm the long-term safety and efficacy of these therapies. As such, personalized treatment remains essential to overcoming the challenges of ARTs. The primary goal is to tailor treatments to the individual biological and clinical characteristics of each patient, thereby improving the likelihood of success. A therapeutic strategy that combines the detection of established and emerging biomarkers, endometrial injury, immunomodulation, and stem cell therapy for regenerating damaged endometrial tissue could offer a practical approach to enhance endometrial receptivity and improve outcomes in assisted reproduction. Overall, endometrial receptivity should be regarded as a systems-level and temporally dynamic biological state rather than a condition definable by individual markers. Future progress in this field will likely depend on integrated multi-omics strategies combined with rigorous prospective clinical validation, shifting the paradigm from single-biomarker approaches toward comprehensive, patient-specific functional profiling of the endometrium.

## Figures and Tables

**Table 2:** Sources and roles of different endometrial biomarkers during implantation process in mammalian species. ER: estrogen receptor, G-CSF: granulocyte colony stimulating factor, IGF: insulin like growth factor, IL: interleukin, KGF: keratinocyte growth factor, LIF: leukemia inhibitory factor, MUC 1: mucin, OPN: osteopontin, PR: progesterone receptor, TGF-β: transforming growth factor beta, VEGF: vascular endothelial growth factor.

Biomarkers	Description	Proposed Roles	Source/Location	Reference
ER	Nuclear receptor for estrogen	Signaling molecules	Endometrial glands and stroma	[77]
PR	Nuclear receptor for progesterone	Signaling molecules	Endometrial glands and stroma	[78]
Pinopods	Progesterone dependent endometrial structures	Signaling and adhesive properties	Surface of the endometrial epithelial cells	[53]
LIF	Pleiotropic cytokine, multifunctional glycoprotein	Signaling	Endometrium	[53]
IL-1	Pro-inflammatory cytokine	Signaling and adhesive roles	Trophoblastic cells and decidualized stromal cells	[79]
IL-6	Multifunctional cytokine	Signaling molecule	Glandular and epithelial cells	[79]
IL-11	Anti-inflammatory cytokine	Signaling Molecule	Endometrium	[45]
IL-15	Cytokine promoting immune cell growth and function	Signaling	Endometrium/ placenta	[46]
OPN	Cytokine known as secreted phosphoprotein 1	Adhesive molecule	Placental and endometrial immune cells	[75]
IGF	Cell growth regulation proteins	Metabolic indicator/ Signaling	Oviduct/ endometrium	[80]
Integrin	Cell adhesion receptor proteins	Adhesive molecule	Endometrium and blastocyst	[76]
MUC 1	Transmembrane glycoprotein	Anti-adhesive molecule	Luminal epithelium of endometrium	[76]
KGF	Implicated in cancer progression	Signaling	Luteal phase of menstrual cycle	[81]
G-CSF	Implicated in implantation success	Signaling	Follicular fluid of endometrium	[79]
VEGF	Critical regulator of blood vessel formation	Signaling	Endothelial cells	[45]
TGF-β	Modulates trophoblast invasion in pregnancy	Signaling	Endometrium	[79]

## Data Availability

No new data were created or analyzed in this study. Data sharing is not applicable to this article.

## References

[1] Li Q., Tsui A.O., Liu L., Ahmed S. (2018). Mortality, Fertility, and Economic Development: An Analysis of 201 Countries from 1960 to 2015. Gates Open Res..

[2] Vander Borght M., Wyns C. (2018). Fertility and Infertility: Definition and Epidemiology. Clin. Biochem..

[3] Agarwal A., Baskaran S., Parekh N., Cho C.L., Henkel R., Vij S., Arafa M., Panner Selvam M.K., Shah R. (2021). Male Infertility. Lancet.

[4] Cimadomo D., de Los Santos M.J., Griesinger G., Lainas G., Le Clef N., McLernon D.J., Montjean D., Toth B., Vermeulen N., ESHRE Working Group on Recurrent Implantation Failure (2023). ESHRE good practice recommendations on recurrent implantation failure. Hum. Reprod. Open..

[5] Carson S.A., Kallen A.N. (2021). Diagnosis and Management of Infertility: A Review. JAMA.

[6] Salamonsen L.A., Hutchison J.C., Gargett C.E. (2021). Cyclical Endometrial Repair and Regeneration. Development.

[7] Large M.J., DeMayo F.J. (2012). The Regulation of Embryo Implantation and Endometrial Decidualization by Progesterone Receptor Signaling. Mol. Cell. Endocrinol..

[8] Siegfried S., Pekonen F., Nyman T., Ammälä M. (1995). Expression of mRNA for Keratinocyte Growth Factor and Its Receptor in Human Endometrium. Acta Obstet. Gynecol. Scand..

[9] Drizi A., Djokovic D., Laganà A.S., van Herendael B. (2020). Impaired Inflammatory State of the Endometrium: A Multifaceted Approach to Endometrial Inflammation. Current Insights and Future Directions. PrzMenopauzalny.

[10] Stuenkel C.A., Gompel A. (2023). Primary Ovarian Insufficiency. N. Engl. J. Med..

[11] Lv M., Yu J., Huang Y., Ma J., Xiang J., Wang Y., Li L., Zhang Z., Liao H. (2022). Androgen Signaling in Uterine Diseases: New Insights and New Targets. Biomolecules.

[12] Craciunas L., Gallos I., Chu J., Bourne T., Quenby S., Brosens J.J., Coomarasamy A. (2019). Conventional and modern markers of endometrial receptivity: A systematic review and meta-analysis. Hum. Reprod. Update.

[13] Maziotis E., Kalampokas T., Giannelou P., Grigoriadis S., Rapani A., Anifantakis M., Kotsifaki A., Pantou A., Triantafyllidou O., Tzanakaki D. (2022). Commercially Available Molecular Approaches to Evaluate Endometrial Receptivity: A Systematic Review and Critical Analysis of the Literature. Diagnostics.

[14] Lee S.K., Kim C.J., Kim D.J., Kang J.H. (2015). Immune Cells in the Female Reproductive Tract. Immune Netw..

[15] Hu X., Wu H., Yong X., Wang Y., Yang S., Fan D., Xiao Y., Che L., Shi K., Li K. (2023). Cyclical Endometrial Repair and Regeneration: Molecular Mechanisms, Diseases, and Therapeutic Interventions. Med. Comm..

[16] Carmichael M.A., Thomson R.L., Moran L.J., Wycherley T.P. (2021). The Impact of Menstrual Cycle Phase on Athletes’ Performance: A Narrative Review. Int. J. Environ. Res. Public Health.

[17] Günther V., Allahqoli L., Deenadayal-Mettler A., Maass N., Mettler L., Gitas G., Andresen K., Schubert M., Ackermann J., von Otte S. (2023). Molecular Determinants of Uterine Receptivity: Comparison of Successful Implantation, Recurrent Miscarriage, and Recurrent Implantation Failure. Int. J. Mol. Sci..

[18] Lessey B.A., Young S.L. (2019). What Exactly Is Endometrial Receptivity?. Fertil. Steril..

[19] Harper M.J. (1992). The Implantation Window. Baillieres Clin. Obstet. Gynaecol..

[20] Enciso M., Aizpurua J., Rodríguez-Estrada B., Jurado I., Ferrández-Rives M., Rodríguez E., Pérez-Larrea E., Climent A.B., Marron K., Sarasa J. (2021). The Precise Determination of the Window of Implantation Significantly Improves ART Outcomes. Sci. Rep..

[21] Do Q.A., Su P.H., Chen C.W., Wang H.C., Lee Y.X., Weng Y.C., Chen L.Y., Hsu Y.H., Lai H.C. (2023). DNA Methylation of Window of Implantation Genes in Cervical Secretions Predicts Ongoing Pregnancy in Infertility Treatment. Int. J. Mol. Sci..

[22] Anoshko Y., Dons’koi B., Sudoma I., Khazhylenko K., Zabara D., Goncharova Y. (2023). Changes in the Immunophenotype of Endometrium During Implantation Window Receptivity Formation in Healthy Fertile Women. Placenta.

[23] Singh N., Sethi A. (2022). Endometritis—Diagnosis,Treatment and its impact on fertility—A Scoping Review. JBRA Assist. Reprod..

[24] Teder H., Koel M., Paluoja P., Jatsenko T., Rekker K., Laisk-Podar T., Kukuškina V., Velthut-Meikas A., Fjodorova O., Peters M. (2018). TAC-seq: Targeted DNA and RNA Sequencing for Precise Biomarker Molecule Counting. npj Genom. Med..

[25] Qiao X., Wu L., Liu D., Pei T., Huang W. (2023). Existence of chronic endometritis and its influence on pregnancy outcomes in infertile women with minimal/mild endometriosis. Int. J. Gynaecol. Obstet..

[26] Pirtea P., Cicinelli E., De Nola R., de Ziegler D., Ayoubi J.M. (2021). Endometrial Causes of Recurrent Pregnancy Losses: Endometriosis, Adenomyosis, and Chronic Endometritis. Fertil. Steril..

[27] Salmeri N., Viganò P., Cavoretto P., Marci R., Candiani M. (2024). The kisspeptin system in and beyond reproduction: Exploring intricate pathways and potential links between endometriosis and polycystic ovary syndrome. Rev. Endocr. Metab. Disord..

[28] Aghajanova L., Stavreus-Evers A., Nikas Y., Hovatta O., Landgren B.M. (2003). Coexpression of Pinopodes and Leukemia Inhibitory Factor, as well as Its Receptor, in Human Endometrium. Fertil. Steril..

[29] Cavagna M., Mantese J.C. (2003). Biomarkers of Endometrial Receptivity—A Review. Placenta.

[30] Parr M.B., Parr E.L. (1974). Uterine Luminal Epithelium: Protrusions Mediate Endocytosis, Not Apocrine Secretion, in the Rat. Biol. Reprod..

[31] Qiong Z., Jie H., Yonggang W., Bin X., Jing Z., Yanping L. (2017). Clinical validation of pinopode as a marker of endometrial receptivity: A randomized controlled trial. Fertil. Steril..

[32] Cohen A.M., Ye X.Y., Colgan T.J., Greenblatt E.M., Chan C. (2020). Comparing Endometrial Receptivity Array to Histologic Dating of the Endometrium in Women with a History of Implantation Failure. Syst. Biol. Reprod. Med..

[33] Zhao L., Yin F., Hu X., Li J., Wei C., Zhou F., Jin X., Tong X., Zhang S.Y. (2025). Pinopode versus endometrial receptivity analysis for personalized embryo transfer in recurrent implantation failure. Reprod. Biomed. Online.

[34] Nikas G. (1999). Pinopodes as Markers of Endometrial Receptivity in Clinical Practice. Hum. Reprod..

[35] Irwin J.C., de las Fuentes L., Giudice L.C. (1994). Growth Factors and Decidualization In Vitro. Ann. N. Y. Acad. Sci..

[36] Cullinan E.B., Abbondanzo S.J., Anderson P.S., Pollard J.W., Lessey B.A., Stewart C.L. (1996). Leukemia Inhibitory Factor (LIF) and LIF Receptor Expression in Human Endometrium Suggests a Potential Autocrine/Paracrine Function in Regulating Embryo Implantation. Proc. Natl. Acad. Sci. USA.

[37] Salleh N., Giribabu N. (2014). Leukemia Inhibitory Factor: Roles in Embryo Implantation and in Nonhormonal Contraception. Sci. World J..

[38] Mikołajczyk M., Skrzypczak J., Szymanowski K., Wirstlein P. (2003). The assessment of LIF in uterine flushing--a possible new diagnostic tool in states of impaired fertility. Reprod. Biol..

[39] Arend W.P., Palmer G., Gabay C. (2008). IL-1, IL-18, and IL-33 Families of Cytokines. Immunol. Rev..

[40] Yoo I., Han J., Kim M., Jang H., Sa S., Choi S.H., Ka H. (2017). Expression and Regulation of Interleukin 6 and Its Receptor at the Maternal-Conceptus Interface during Pregnancy in Pigs. Theriogenology.

[41] Mardanian F., Sheikh-Soleimani Z. (2014). The diagnostic role of cervico-vaginal fluid interleukins-1α in endometriosis: A case-control study. J. Res. Med. Sci..

[42] Khadem N., Mansoori M., Attaran M., Attaranzadeh A., Zohdi E. (2019). Association of IL-1 and TNF-α Levels in Endometrial Secretion and Success of Embryo Transfer in IVF/ICSI Cycles. Int. J. Fertil. Steril..

[43] Nishino E., Matsuzaki N., Masuhiro K., Kameda T., Taniguchi T., Takagi T., Saji F., Tanizawa O. (1990). Trophoblast-Derived Interleukin-6 (IL-6) Regulates Human Chorionic Gonadotropin Release through IL-6 Receptor on Human Trophoblasts. J. Clin. Endocrinol. Metab..

[44] Sherwin J.R., Smith S.K., Wilson A., Sharkey A.M. (2002). Soluble gp130 Is Up-Regulated in the Implantation Window and Shows Altered Secretion in Patients with Primary Unexplained Infertility. J. Clin. Endocrinol. Metab..

[45] vanMourik M.S., Macklon N.S., Heijnen C.J. (2009). Embryonic Implantation: Cytokines, Adhesion Molecules, and Immune Cells in Establishing an Implantation Environment. J. Leukoc. Biol..

[46] Kokot I., Piwowar A., Jędryka M., Sołkiewicz K., Kratz E.M. (2021). Diagnostic Significance of Selected Serum Inflammatory Markers in Women with Advanced Endometriosis. Int. J. Mol. Sci..

[47] Du X.X., Williams D.A. (1994). Interleukin-11: A Multifunctional Growth Factor Derived from the Hematopoietic Microenvironment. Blood.

[48] Guzeloglu-Kayisli O., Kayisli U.A., Taylor H.S. (2009). The Role of Growth Factors and Cytokines during Implantation: Endocrine and Paracrine Interactions. Semin. Reprod. Med..

[49] Okada H., Nakajima T., Yasuda K., Kanzaki H. (2004). Interleukin-1 Inhibits Interleukin-15 Production by Progesterone during In Vitro Decidualization in Humans. J. Reprod. Immunol..

[50] Kurmanova A., Ashirbekov Y., Kurmanova G., Mamedaliyeva N., Anartayeva G., Moshkalova G., Salimbayeva D., Tulesheva A., Zhankina Z. (2024). Altered Expressions of *IL-15*, *IFNG*, and *HPRT1* Genes in the Thin Endometria of Patients with Reproductive Disorders: A Prospective Comparative Study. J. Clin. Med..

[51] Dey S.K., Lim H., Das S.K., Reese J., Paria B.C., Daikoku T., Wang H. (2004). Molecular Cues to Implantation. Endocr. Rev..

[52] Bass K.E., Morrish D., Roth I., Bhardwaj D., Taylor R., Zhou Y., Fisher S.J. (1994). Human Cytotrophoblast Invasion Is Up-Regulated by Epidermal Growth Factor: Evidence That Paracrine Factors Modify This Process. Dev. Biol..

[53] Kliman H.J., Honig S., Walls D., Luna M., McSweet J.C., Copperman A.B. (2006). Optimization of Endometrial Preparation Results in a Normal Endometrial Function Test (EFT) and Good Reproductive Outcome in Donor Ovum Recipients. J. Assist. Reprod. Genet..

[54] Camargo-Díaz F., García V., Ocampo-Bárcenas A., González-Marquez H., López-Bayghen E. (2017). Colony stimulating factor-1 and leukemia inhibitor factor expression from current-cycle cannula isolated endometrial cells are associated with increased endometrial receptivity and pregnancy. BMC Women’s Health.

[55] Simón C., Martín J.C., Pellicer A. (2000). Paracrine Regulators of Implantation. Baillieres Best Pract. Res. Clin. Obstet. Gynaecol..

[56] Leach R.E., Khalifa R., Ramirez N.D., Das S.K., Wang J., Dey S.K., Romero R., Armant D.R. (1999). Multiple Roles for Heparin-Binding Epidermal Growth Factor-Like Growth Factor Are Suggested by Its Cell-Specific Expression during the Human Endometrial Cycle and Early Placentation. J. Clin. Endocrinol. Metab..

[57] Martin K.L., Barlow D.H., Sargent I.L. (1998). Heparin-Binding Epidermal Growth Factor Significantly Improves Human Blastocyst Development and Hatching in Serum-Free Medium. Hum. Reprod..

[58] Tabibzadeh S., Hemmati-Brivanlou A. (2006). Lefty at the Crossroads of "Stemness" and Differentiative Events. Stem Cells.

[59] Robertson S.A., Sharkey D.J. (2016). Seminal fluid and fertility in women. Fertil. Steril..

[60] Sharkey D.J., Tremellen K.P., Briggs N.E., Dekker G.A., Robertson S.A. (2016). Seminal plasma transforming growth factor-β, activin A and follistatin fluctuate within men over time. Hum. Reprod..

[61] Latifi Z., Nejabati H.R., Abroon S., Mihanfar A., Farzadi L., Hakimi P., Hajipour H., Nouri M., Fattahi A. (2019). Dual role of TGF-β in early pregnancy: Clues from tumor progression. Biol. Reprod..

[62] Bischof P., Campana A. (2000). Molecular Mediators of Implantation. Baillieres Best Pract. Res. Clin. Obstet. Gynaecol..

[63] Mazella J., Tang M., Tseng L. (2004). Disparate Effects of Relaxin and TGFbeta1: Relaxin Increases, but TGFbeta1 Inhibits, the Relaxin Receptor and the Production of IGFBP-1 in Human Endometrial Stromal/Decidual Cells. Hum. Reprod..

[64] Giudice L.C., Irwin J.C. (1999). Roles of the Insulin-like Growth Factor Family in Nonpregnant Human Endometrium and at the Decidual: Trophoblast Interface. Semin. Reprod. Endocrinol..

[65] Fazleabas A.T., Kim J.J., Srinivasan S., Donnelly K.M., Brudney A., Jaffe R.C. (1999). Implantation in the Baboon: Endometrial Responses. Semin. Reprod. Endocrinol..

[66] Fowler D.J., Nicolaides K.H., Miell J.P. (2000). Insulin-Like Growth Factor Binding Protein-1 (IGFBP-1): A Multifunctional Role in the Human Female Reproductive Tract. Hum. Reprod. Update.

[67] Xu H., Xie Z., Zheng Y., Wang C. (2026). The predictive diagnostic value of combined serum detection of CXCL12, VEGF, and CA125 for endometriosis. Medicine.

[68] Olofsson B., Korpelainen E., Pepper M.S., Mandriota S.J., Aase K., Kumar V., Gunji Y., Jeltsch M.M., Shibuya M., Alitalo K. (1998). Vascular Endothelial Growth Factor B (VEGF-B) Binds to VEGF Receptor-1 and Regulates Plasminogen Activator Activity in Endothelial Cells. Proc. Natl. Acad. Sci. USA.

[69] Fu L.L., Xu Y., Yan J., Zhang X.Y., Li D.D., Zheng L.W. (2023). Efficacy of Granulocyte Colony-Stimulating Factor for Infertility Undergoing IVF: A Systematic Review and Meta-Analysis. Reprod. Biol. Endocrinol..

[70] Guo X., Yi H., Li T.C., Wang Y., Wang H., Chen X. (2021). Role of Vascular Endothelial Growth Factor (VEGF) in Human Embryo Implantation: Clinical Implications. Biomolecules.

[71] Rubin J.S., Osada H., Finch P.W., Taylor W.G., Rudikoff S., Aaronson S.A. (1989). Purification and Characterization of a Newly Identified Growth Factor Specific for Epithelial Cells. Proc. Natl. Acad. Sci. USA.

[72] Larsen M.H., Bzorek M., Pass M.B., Larsen L.G., Nielsen M.W., Svendsen S.G., Lindhard A., Hviid T.V. (2011). Human leukocyte antigen-G in the male reproductive system and in seminal plasma. Mol. Hum. Reprod..

[73] Schallmoser A., Raab M., Karn T., Königsberger S., Schmidt E., Breitenbach-Koller H., Sänger N. (2019). Quantitative analysis of the sHLA-G protein in seminal plasma. Am. J. Reprod. Immunol..

[74] Lessey B.A., Castelbaum A.J., Sawin S.W., Sun J. (1995). Integrins as markers of uterine receptivity in women with primary unexplained infertility. Fertil. Steril..

[75] Ishikawa A., Kudo M., Nakazawa N., Onda M., Ishiwata T., Takeshita T., Naito Z. (2008). Expression of Keratinocyte Growth Factor and Its Receptor in Human Endometrial Cancer in Cooperation with Steroid Hormones. Int. J. Oncol..

[76] Raheem K.A. (2017). Cytokines, Growth Factors and Macromolecules as Mediators of Implantation in Mammalian Species. Int. J. Vet. Sci. Med..

[77] Cui J., Shen Y., Li R. (2013). Estrogen Synthesis and Signaling Pathways During Aging: From Periphery to Brain. Trends Mol. Med..

[78] Bourgain C., Devroey P., Van Waesberghe L., Smitz J., Van Steirteghem A.C. (1990). Effects of Natural Progesterone on the Morphology of the Endometrium in Patients with Primary Ovarian Failure. Hum. Reprod..

[79] Koopman L.A., Kopcow H.D., Rybalov B., Boyson J.E., Orange J.S., Schatz F., Masch R., Lockwood C.J., Schachter A.D., Park P.J. (2003). Human Decidual Natural Killer Cells Are a Unique NK Cell Subset with Immunomodulatory Potential. J. Exp. Med..

[80] Fatmous M., Rai A., Poh Q.H., Salamonsen L.A., Greening D.W. (2022). Endometrial Small Extracellular Vesicles Regulate Human Trophectodermal Cell Invasion by Reprogramming the Phosphoproteome Landscape. Front. Cell Dev. Biol..

[81] Finch P.W., Rubin J.S. (2006). Keratinocyte Growth Factor Expression and Activity in Cancer: Implications for Use in Patients with Solid Tumors. J. Natl. Cancer Inst..

[82] King A., Burrows T., Loke Y.W. (1996). Human uterine natural killer cells. Nat. Immunol..

[83] Garratt J., Rahmati M. (2023). Assessing the Endometrium: An Update on Current and Potential Novel Biomarkers of Receptivity. J. Reprod. Immunol..

[84] Diaz-Martínez M.D.C., Bernabeu A., Lledó B., Carratalá-Munuera C., Quesada J.A., Lozano F.M., Ruiz V., Morales R., Llácer J., Ten J. (2021). Impact of the Vaginal and Endometrial Microbiome Pattern on Assisted Reproduction Outcomes. J. Clin. Med..

[85] Moreno I., Codoñer F.M., Vilella F., Valbuena D., Martinez-Blanch J.F., Jimenez-Almazán J., Alonso R., Alamá P., Remohí J., Pellicer A. (2016). Evidence that the Endometrial Microbiota Has an Effect on Implantation Success or Failure. Am. J. Obstet. Gynecol..

[86] Li Q., Liu W., Chiu P.C.N., Yeung W.S.B. (2020). miR-let-7a/g Enhances Uterine Receptivity via Suppressing Wnt/β-Catenin under the Modulation of Ovarian Hormones. Reprod. Sci..

[87] Southcombe J., Tannetta D., Redman C., Sargent I. (2011). The Immunomodulatory Role of Syncytiotrophoblast Microvesicles. PLoS ONE.

[88] Menkhorst E.M., Gamage T., Cuman C., Kaitu’u-Lino T.J., Tong S., Dimitriadis E. (2014). Galectin-7 Acts as an Adhesion Molecule During Implantation and Increased Expression Is Associated with Miscarriage. Placenta.

[89] Yu M., Wang J., Liu S., Wang X., Yan Q. (2017). Novel Function of Pregnancy-Associated Plasma Protein A: Promotes Endometrium Receptivity by Up-Regulating N-Fucosylation. Sci. Rep..

[90] Taylor H.S., VandenHeuvel G.B., Igarashi P. (1997). A Conserved Hox Axis in the Mouse and Human Female Reproductive System: Late Establishment and Persistent Adult Expression of the Hoxa Cluster Genes. Biol. Reprod..

[91] Vitiello D., Kodaman P.H., Taylor H.S. (2007). HOX Genes in Implantation. Semin. Reprod. Med..

[92] Haouzi D., Mahmoud K., Fourar M., Bendhaou K., Dechaud H., De Vos J., Rème T., Dewailly D., Hamamah S. (2009). Identification of New Biomarkers of Human Endometrial Receptivity in the Natural Cycle. Hum. Reprod..

[93] Kicińska A.M., Maksym R.B., Zabielska-Kaczorowska M.A., Stachowska A., Babińska A. (2023). Immunological and Metabolic Causes of Infertility in Polycystic Ovary Syndrome. Biomedicines.

[94] Doyle N., Jahandideh S., Hill M.J., Widra E.A., Levy M., Devine K. (2022). Effect of Timing by Endometrial Receptivity Testing vs Standard Timing of Frozen Embryo Transfer on Live Birth in Patients Undergoing In Vitro Fertilization: A Randomized Clinical Trial. JAMA.

[95] Hamamah S., Haouzi D. (2016). Methods for Assessing Endometrium Receptivity of a Patient. https://patents.google.com/patent/US9260748B2/en.

[96] Rubin S.C., Abdulkadir M., Lewis J., Harutyunyan A., Hirani R., Grimes C.L. (2023). Review of Endometrial Receptivity Array: A Personalized Approach to Embryo Transfer and Its Clinical Applications. J. Pers. Med..

[97] Meltsov A., Saare M., Teder H., Paluoja P., Arffman R.K., Piltonen T., Laudanski P., Wielgoś M., Gianaroli L., Koel M. (2023). Targeted gene expression profiling for accurate endometrial receptivity testing. Sci. Rep..

[98] Haouzi D., Entezami F., Torre A., Innocenti C., Antoine Y., Mauries C., Vincens C., Bringer-Deutsch S., Gala A., Ferrieres-Hoa A. (2021). Customized Frozen Embryo Transfer after Identification of the Receptivity Window with a Transcriptomic Approach Improves the Implantation and Live Birth Rates in Patients with Repeated Implantation Failure. Reprod. Sci..

[99] Lensen S.F., Armstrong S., Gibreel A., Nastri C.O., Raine-Fenning N., Martins W.P. (2021). Endometrial Injury in Women Undergoing In Vitro Fertilisation (IVF). Cochrane Database Syst. Rev..

[100] Siristatidis C., Vrachnis N., Vogiatzi P., Chrelias C., Retamar A.Q., Bettocchi S., Glujovsky D. (2014). Potential Pathophysiological Mechanisms of the Beneficial Role of Endometrial Injury in In Vitro Fertilization Outcome. Reprod. Sci..

[101] Kalma Y., Granot I., Gnainsky Y., Or Y., Czernobilsky B., Dekel N., Barash A. (2009). Endometrial Biopsy-Induced Gene Modulation: First Evidence for the Expression of Bladder-TransmembranalUroplakinIb in Human Endometrium. Fertil. Steril..

[102] Pourmoghadam Z., Soltani-Zangbar M.S., Sheikhansari G., Azizi R., Eghbal-Fard S., Mohammadi H., Siahmansouri H., Aghebati-Maleki L., Danaii S., Mehdizadeh A. (2020). Intrauterine Administration of Autologous hCG-Activated Peripheral Blood Mononuclear Cells Improves Pregnancy Outcomes in Patients with Recurrent Implantation Failure; A Double-Blind, Randomized Control Trial Study. J. Reprod. Immunol..

[103] Schallmoser A., Emrich N., Masslow L., Allam J.P., Einenkel R., Sänger N. (2025). HLA-G, IL-6 and TGF-β in Seminal Plasma as Potential Biomarkers of ART Outcome. Am. J. Reprod. Immunol..

[104] van den Berg J.S., Toros M., Abendroth M.S., Zhang Y., Arends B., Verpoest W.M.J.A., Broekmans F.J.M., Steba G.S. (2025). Seminal plasma induces receptivity-associated genes and pathways in endometrial epithelial organoids. Reprod. Biomed. Online.

[105] Song J., Li M., Tao Y., Li Y., Mai C., Zhang J., Yao L., Shi S., Xu J. (2025). Enhanced myofibroblast differentiation of eMSCs in intrauterine adhesions. Stem Cell Res. Ther..

[106] Wu X., Jiang J., Gu Z., Zhang J., Chen Y., Liu X. (2020). Mesenchymal stromal cell therapies: Immunomodulatory properties and clinical progress. Stem Cell Res. Ther..

